# Cancer Chemopreventive Effect of 2′,4′-Dihydroxy-6′-methoxy-3′,5′-dimethylchalcone on Diethylnitrosamine-Induced Early Stages of Hepatocarcinogenesis in Rats

**DOI:** 10.3390/plants13141975

**Published:** 2024-07-19

**Authors:** Sirinya Taya, Charatda Punvittayagul, Puttinan Meepowpan, Rawiwan Wongpoomchai

**Affiliations:** 1 Functional Food Research Unit, Multidisciplinary Research Institute, Chiang Mai University, Chiang Mai 50200, Thailand; rawiwan.wong@cmu.ac.th; 2 Center of Veterinary Medical Diagnostic and Animal Health Innovation, Faculty of Veterinary Medicine, Chiang Mai University, Chiang Mai 50100, Thailand; charatda.pun@cmu.ac.th; 3 Department of Chemistry, Faculty of Science, Chiang Mai University, Chiang Mai 50200, Thailand; pmeepowpan@gmail.com; 4 Department of Biochemistry, Faculty of Medicine, Chiang Mai University, Chiang Mai 50200, Thailand

**Keywords:** 2′,4′-dihydroxy-6′-methoxy-3′,5′-dimethylchalcone, *Cleistocalyx nervosum* var. *paniala* seed, cancer chemoprevention, hepatocarcinogenesis

## Abstract

2′,4′-dihydroxy-6′-methoxy-3′,5′-dimethylchalcone (DMC) is a major compound in *Cleistocalyx nervosum* seed extract (CSE), which has been reported to have various biological activities, including anti-cancer activity. Therefore, this study attempted to evaluate whether DMC is a chemopreventive compound in CSE. Moreover, the preventive mechanisms of CSE and DMC in the DEN-induced early stages of hepatocarcinogenesis in rats were investigated. Male Wistar rats were intraperitoneally injected with DEN 50 mg/kg bw once a week for 8 weeks. Rats received CSE and DMC orally throughout the experiment. The number of glutathione *S*-transferase placental form (GST-P)-positive foci in the liver was measured. Furthermore, the preventive mechanisms of CSE and DMC on DEN-induced HCC, including cell proliferation and apoptosis, were investigated. Administering CSE at a dosage of 400 mg/kg bw and DMC at a dosage of 10 mg/kg bw significantly decreased the number and size of GST-P-positive foci and GST-P expression. In addition, DMC inhibited the development of preneoplastic lesions by decreasing cell proliferation and causing cell apoptosis; however, CSE inhibited the development of preneoplastic lesions by inducing cell apoptosis. In conclusion, DMC exhibited a cancer chemopreventive effect on the early stages of hepatocarcinogenesis by increasing cell apoptosis and reducing cell proliferation.

## 1. Introduction

Hepatocellular carcinoma (HCC) is the sixth most diagnosed cancer and the third leading cause of cancer-related death worldwide [1]. Nowadays, there are various HCC treatments, including surgery, liver transplantation, radiation therapy, chemotherapy, immunotherapy, and gene therapy [2]. However, the limitations of treatment strategies for HCC are mainly based on cancer stages, side effects, and unaffordable cancer care [2,3,4]. Due to the limitations of cancer treatments, cancer preventive strategies have been considered a suitable approach for preventing or delaying HCC incidence.

Cancer chemoprevention is the use of natural and synthetic compounds to suppress, prevent, or delay carcinogenesis by interfering with the initiation and/or promotion stages [5]. Exploring the cancer chemopreventive potential of herbs, vegetables, fruits, and plants, as well as natural products, has attracted a lot of attention. Additionally, trends in research suggest an interest in using natural product constituents instead of crude extracts to prevent the adverse effects of other constituents [6].

*Cleistocalyx nervosum* var. *paniala* is a local fruit that grows in the northern provinces of Thailand. *C. nervosum* seeds, waste products from fruit-processing production, contain phenolic compounds, flavonoids, and condensed tannins [7]. Our studies reported that the methanolic extract of *C. nervosum* seed presented antigenotoxicity in a *Salmonella* mutation assay and a rat liver micronucleus test [7]. Furthermore, the dichloromethane extract of *C. nervosum* seed inhibited diethylnitrosamine (DEN)-induced rat micronucleated formation and exhibited chemopreventive potential against chemically induced liver and colon carcinogenesis in rats [8]. The main flavonoid in the dichloromethane extract of *C. nervosum* seed is 2′,4′-dihydroxy-6′-methoxy-3′,5′-dimethylchalcone (DMC; C_18_H_18_O_4_) [8]. Several studies have documented the biological activities of DMC, which include anti-inflammatory, hepatoprotective, and especially anticancer properties [9,10,11,12,13]. A study by Vachiraarunwong et al. also found that DMC from *C. nervosum* seed had a chemopreventive effect on the post-initiation stage of colorectal carcinogenesis by inducing xenobiotic metabolizing enzymes and suppressing cell proliferation [14]. Moreover, DMC isolated from the buds of *C*. *operculatus* had antitumor activity *in vivo* by using a solid human tumor xenograft model with a human liver cancer SMMC-7721 cell line [15]. However, the cancer chemopreventive properties of DMC on chemical-induced hepatocarcinogenesis in animal models have not yet been thoroughly investigated. Diethylnitrosamine (DEN) is a potent hepatocarcinogen that is used to induce HCC in rodent experimental models [16,17]. DEN has been found in various products, including tobacco smoke, cured meat products, and processed fish [18,19]. Moreover, DEN-induced hepatocarcinogenesis in rats is one of the animal models that mimics human liver carcinogenesis at different stages of HCC. This model has been widely used to evaluate the chemopreventive or anticancer potency of phytochemicals or crude extracts [20,21,22]. Therefore, this work aims to evaluate whether DMC is a chemopreventive compound in CSE. Moreover, the inhibitory mechanisms of *C. nervosum* seed extract (CSE) and DMC against the early stages of hepatocarcinogenesis induced by DEN in rats were investigated.

## 2. Results

### 2.1. Effects of CSE and DMC on General Observations

General observations, such as body weights, diet and water consumption, and relative organ weights of rats (details of each experimental group are fully described in Section 4.3) treated with the CSE and the DMC are presented in Table 1 and Table 2. Rats injected with DEN showed a significant decrease in final body weight and diet intake when compared to normal saline solution (NSS)-treated rats. Nevertheless, administrations of the CSE and the DMC to rats treated with DEN did not result in any significant change in the final body weight and diet intake compared to rats treated with DEN alone. Furthermore, the final body weight and diet intake of the NSS-treated rats significantly decreased after the administration of CSE at a dosage of 400 mg/kg bw. Nevertheless, administering DMC did not have any impact on the final body weight and the intake of diet and water in rats treated with NSS. Throughout the experiment, there was no difference in water consumption across the groups. However, the treatments of the CSE at 200 mg/kg bw and the DMC at 20 mg/kg bw in DEN-treated rats resulted in a significant increase in water intake when compared to DEN-treated rats. Relative liver, spleen, and kidney weights showed no significant difference across the groups. Nevertheless, CSE treatment at a dosage of 400 mg/kg bw in rats treated with DEN or NSS led to a significant increase in the relative weight of the kidney as compared to their corresponding control groups (Table 2). These findings indicated that DMC had no adverse effect on the growth of the rats and did not present any toxicity when administered to rats. However, feeding CSE at a dosage of 400 mg/kg bw affected the growth of the rats and might be toxic to the rats.

### 2.2. Effects of CSE and DMC on Serum Biochemical Parameters

**Figure 1:**
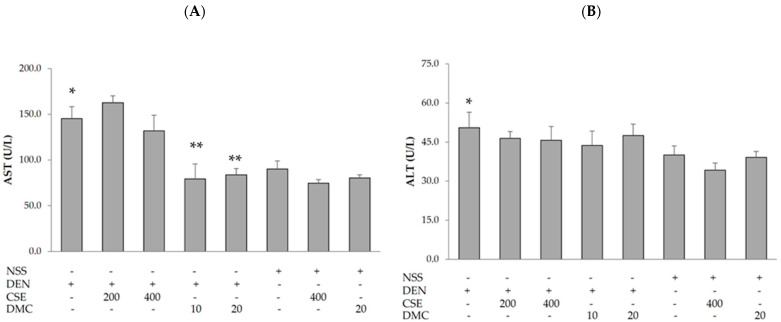
Effects of CSE and DMC on levels of (**A**) AST and (**B**) ALT in rats. The results are presented as mean ± SD. Diethylnitrosamine (DEN); normal saline solution (NSS); *C. nervosum* seed extract (CSE); 2′,4′-dihydroxy-6′-methoxy-3′,5′-dimethylchalcone (DMC). * Significantly different compared to the NSS-treated group (*p* < 0.05). ** Significantly different compared to the DEN-treated group (*p* < 0.05).

Hepatic damage is assessed by liver function tests such as alanine transaminase (ALT), aspartate transaminase (AST), total protein, albumin, total bilirubin, and direct bilirubin. The elevated pattern in these tests might assist in organizing a differential diagnosis [23]. The effects of CSE and DMC on the liver enzymes, AST and ALT, in rats are presented in Figure 1. The administration of DEN showed a significant increase in the levels of AST and ALT when compared to NSS-treated rats. Moreover, treatments with DMC at 10 and 20 mg/kg bw in DEN-treated rats (79.3 ± 16.5 and 83.7 ± 7.2 U/L) resulted in a significant decrease in the level of AST when compared to DEN-treated rats (145.3 ± 12.9 U/L). Nevertheless, administering the CSE and the DMC to rats treated with DEN did not result in any significant change in the level of ALT compared to rats treated with DEN alone. Interestingly, administering the CSE and the DMC at high doses to rats treated with NSS had no significant effect on AST and ALT levels. Additionally, the effects of CSE and DMC on total protein, albumin, total bilirubin, and direct bilirubin in rats are summarized in Table 3. In comparison to rats treated with NSS, the injection of DEN resulted in a statistically significant increase in direct bilirubin level. At dosages of 10 and 20 mg/kg bw, DMC showed a significant decline in the level of direct bilirubin. However, there were no significant differences in total protein, albumin, and total bilirubin levels as observed in DEN-treated rats when compared to NSS-treated rats. Furthermore, treatments of the CSE and the DMC in rats treated with DEN did not alter these parameters, total protein, albumin, and total bilirubin levels, when compared to DEN-treated rats. Remarkably, the administration of a high dose of CSE resulted in a significant increase in albumin level, while the administration of a low dose of DMC led to a significant decrease in the level of total bilirubin in rats treated with DEN as compared to rats treated with DEN alone. High doses of the CSE and the DMC administered to rats treated with NSS had no significant impact on total protein, albumin, total bilirubin, or direct bilirubin levels; however, DMC significantly decreased albumin levels. Nevertheless, this change was within the normal range of reference data [24]. These findings revealed that DMC protected against DEN-induced liver damage.

Because a high dose of CSE significantly increased relative kidney weight in DEN- or NSS-treated rats, serum renal markers, including blood urea nitrogen (BUN) and creatinine, were examined. There was a significant elevation in the level of BUN in DEN-treated rats when compared with NSS-treated rats. Moreover, the level of creatinine tended to increase in DEN-treated rats (Table 3). Administering DMC at a dose of 10 mg/kg bw to rats treated with DEN resulted in significant reductions in BUN and creatinine levels compared to rats treated with DEN alone. Interestingly, high doses of the CSE and the DMC administered to rats treated with NSS had no significant impact on levels of BUN and creatinine (Table 3). These findings indicated that DMC protected against DEN-induced renal damage, while CSE did not induce renal injury in NSS-treated rats.

### 2.3. Effects of CSE and DMC on Histopathological Lesions in Rat Liver

Table 4 summarizes the histopathological lesions discovered in the rat livers. There was a significant increase in the number of focal hepatocellular hyperplasia in DEN-treated rats when compared with NSS-treated rats. Moreover, administering CSE at a dose of 400 mg/kg bw to rats treated with DEN resulted in a significant reduction in the number of focal hepatocellular hyperplasia compared to rats treated with DEN alone. However, the numbers of hepatocellular adenoma and hepatocellular carcinoma showed no significant differences across the groups.

### 2.4. Effects of CSE and DMC on Preneoplastic Lesion in Rat Liver

**Figure 2:**
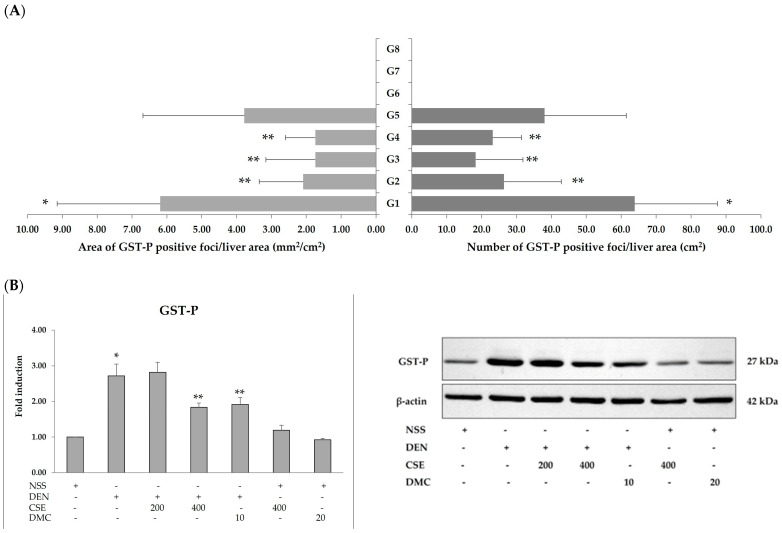
Effects of CSE and DMC on glutathione *S*-transferase placental form (GST-P)-positive foci expression in rat liver. Diethylnitrosamine (DEN); normal saline solution (NSS); *C. nervosum* seed extract (CSE); 2′,4′-dihydroxy-6′-methoxy-3′,5′-dimethylchalcone (DMC). (**A**) Immunohistochemistry; (**B**) Western blot analysis. Values are expressed as the mean ± SD. G1 through G8 are fully described in Section 4.3. * Significantly different compared to the NSS-treated group (*p* < 0.05). ** Significantly different compared to the DEN-treated group (*p* < 0.05).

The effects of CSE and DMC on preneoplastic lesions, GST-P-positive foci, was examined by immunohistochemistry. Rats treated with DEN showed a significant increase in both the area and number of GST-P-positive foci in comparison to rats treated with NSS. Moreover, administering CSE at doses of 200 (2.09 ± 1.26 and 26.44 ± 16.40) and 400 mg/kg bw (1.74 ± 1.42 and 18.30 ± 13.51) and DMC at a dose of 10 mg/kg bw (1.74 ± 0.86 and 23.17 ± 8.23) to rats treated with DEN resulted in significant decreases in the area and number of GST-P-positive foci compared to rats treated with DEN alone (6.19 ± 2.96 and 63.85 ± 23.71) (Figure 2A). Interestingly, CSE and DMC at high doses did not induce GST-P-positive foci in NSS-treated rats. Furthermore, the effects of CSE and DMC on GST-P expression in rat liver were confirmed by Western blot analysis. The administration of DEN showed a significant increase in GST-P expression when compared to NSS-treated rats. In addition, administering CSE at a dosage of 400 mg/kg bw and DMC at a dosage of 10 mg/kg bw to rats treated with DEN resulted in a significant reduction in GST-P expression, as shown in Figure 2B. These findings indicated that CSE and DMC had no carcinogenic effect on the rat liver. Furthermore, a high dose of CSE and a low dose of DMC attenuated preneoplastic lesions in the rat liver.

### 2.5. Effects of CSE and DMC on Cell Proliferation and Apoptosis in Rat Liver

**Figure 3:**
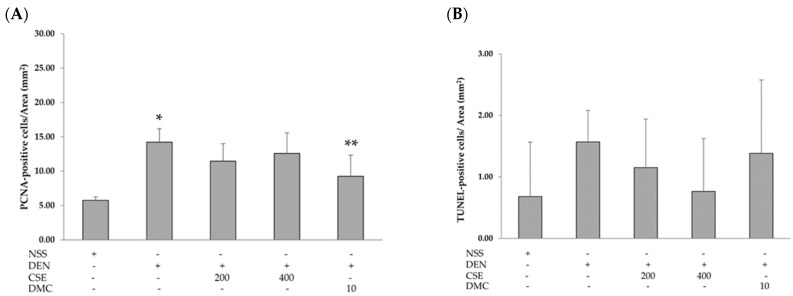
Effects of CSE and DMC on cell proliferation and apoptosis in rat liver. The number of (**A**) PCNA-positive and (**B**) TUNEL-positive cells in rat liver. Values are expressed as the mean ± SD. Diethylnitrosamine (DEN); normal saline solution (NSS); *C. nervosum* seed extract (CSE); 2′,4′-dihydroxy-6′-methoxy-3′,5′-dimethylchalcone (DMC). * Significantly different compared to the NSS-treated group (*p* < 0.05). ** Significantly different compared to the DEN-treated group (*p* < 0.05).

**Figure 4:**
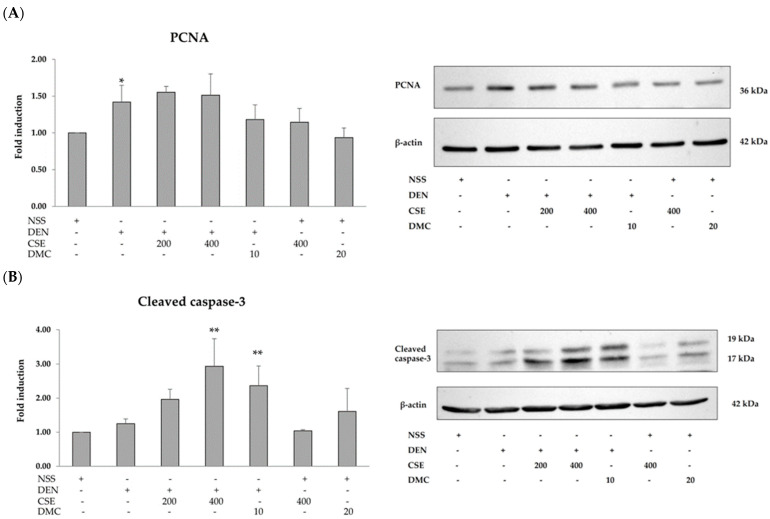
Effects of CSE and DMC on the expression of proteins associated with proliferation and apoptosis in rat liver. (**A**) PCNA and (**B**) cleaved caspase-3. Values are expressed as the mean ± SD. Diethylnitrosamine (DEN); normal saline solution (NSS); *C. nervosum* seed extract (CSE); 2′,4′-dihydroxy-6′-methoxy-3′,5′-dimethylchalcone (DMC). * Significantly different compared to the NSS-treated group (*p* < 0.05). ** Significantly different compared to the DEN-treated group (*p* < 0.05).

Because CSE and DMC dramatically reduced GST-P-positive foci in DEN-treated rats, their possible mechanisms of cell proliferation and apoptosis were investigated. There was a significant increase in the number of PCNA-positive cells in DEN-treated rats (14.23 ± 1.94) when compared with NSS-treated rats (5.76 ± 0.51). Furthermore, administering DMC 10 mg/kg bw to rats treated with DEN (9.26 ± 3.08) markedly reduced the number of PCNA-positive cells compared to rats treated with DEN alone (14.23 ± 1.94) (Figure 3A). However, the number of TUNEL-positive cells showed no significant differences across the groups (Figure 3B). The effects of CSE and DMC on the expression of proteins involved in cell proliferation and apoptosis in the rat liver are presented in Figure 4. PCNA expression in DEN-treated rats showed a significant increase compared to NSS-treated rats. Furthermore, administering DMC at a dosage of 10 mg/kg bw to rats treated with DEN resulted in a tendency to decrease PCNA expression compared to rats treated with DEN alone (Figure 4A). However, there was no significant change in cleaved caspase-3 expression between rats treated with DEN and those treated with NSS. Surprisingly, CSE at 400 mg/kg bw and DMC at 10 mg/kg bw resulted in a significant increase in cleaved caspase-3 expression in rats treated with DEN compared to rats treated with DEN alone (Figure 4B). According to these results, DMC inhibited the development of preneoplastic lesions in DEN-initiated rats by decreasing cell proliferation and causing cell apoptosis; on the other hand, CSE inhibited the development of preneoplastic lesions in DEN-initiated rats by inducing cell apoptosis.

## 3. Discussion

According to our previous published findings, administering 200 mg/kg bw of CSE had a chemopreventive effect on chemically induced carcinogenesis in both the initiation and promotion stages [8]. DMC was one of the chemopreventive compounds obtained from CSE. Nevertheless, administering DMC at a dosage of 20 mg/kg bw, which is equivalent to 400 mg/kg bw of CSE, displayed a chemopreventive effect on DEN- and DMH-induced colorectal carcinogenesis but did not have any effect on hepatocarcinogenesis. Thus, this study used DEN-induced hepatocarcinogenesis, an animal model that mimics the development of liver cancer in humans at different stages of HCC, to evaluate the chemopreventive effects of CSE and DMC at 200 and 400 mg/kg bw of CSE and DMC at a dosage equivalent to that found in CSE on the early stages of hepatocarcinogenesis in rats. Our findings revealed that both CSE and DMC exhibited a chemopreventive effect on the early stages of DEN-induced hepatocarcinogenesis. The administrations of CSE at a dosage of 400 mg/kg bw and DMC at a dosage of 10 mg/kg bw significantly decreased preneoplastic lesions. Furthermore, administering CSE at a dosage of 400 mg/kg bw significantly reduced the incidence of focal hepatocellular hyperplasia caused by DEN. Similarly, DMC at a dose of 10 mg/kg bw showed a tendency to lower the number of focal hepatocellular hyperplasia induced by DEN. On the other hand, Ye et al. found that DMC has anticancer efficacy in a solid human carcinoma xenograft model [15]. It is interesting to note that neither DMC nor CSE caused the formation of GST-P-positive foci, focal hepatocellular hyperplasia, hepatocellular adenoma, or hepatocellular carcinoma in rats, indicating that they are not carcinogenic. The liver function tests were used to assess the hepatic damage. In the present study, the DEN-treated rats exhibited increased levels of serum AST, ALT, and direct bilirubin. These raised serum levels suggested injury and death to the hepatocytes [25]. Furthermore, severe hepatic parenchymal damage is associated with hyperbilirubinemia, which indicates that the liver is unable to conjugate and excrete bilirubin [26]. However, administering DMC restored the levels of AST and direct bilirubin in DEN-treated rats. It could be inferred that DMC prevents liver damage induced by DEN. According to Yu et al., DMC showed a protective effect against acute hepatotoxicity induced by CCl_4_ by reducing oxidative stress, enhancing the antioxidative cascade, and inhibiting lipid peroxidation in the liver [13]. However, administering CSE at a dosage of 400 mg/kg bw resulted in a decrease in body weight and diet consumption, as well as an increase in relative kidney weight, although the DMC treatment did not affect those parameters. It might be implied that treatment with a high dose of CSE causes adverse effects, which is consistent with our previous findings. It was found that 71 compounds were discovered in CSE by GC-MS, and of those, 16 identified compounds were phytosterols and terpenoids [8], probably leading to those effects when amalgamated. Currently, there has been several studies on the pure compounds from phytochemicals as an alternative to crude extracts, due to their adverse effects [6]. These data suggested that DMC rather than CSE could be a potential candidate for inhibiting DEN-induced HCC, as demonstrated by improved serum liver function indicators, the presence of GST-P-positive foci, and focal hepatocellular hyperplasia. Additionally, it may be less toxic than CSE.

Cancer is characterized by uncontrolled and abnormal cell growth. Apoptosis evasion is one of the hallmarks of malignancies that contribute to tumor formation and progression, as well as unregulated cell proliferation. Most anticancer drugs act by directly activating the apoptotic mechanisms in cancer cells. However, natural products or their derivatives have attracted more attention in the treatment of cancer than other categories of anticancer drugs due to their abundance in nature and their minimal to lacking adverse effects. Based on this study, the administrations of the CSE and the DMC led to a decrease in the development of early stages of hepatocarcinogenesis, as shown by a reduction in the formation of GST-P-positive foci. The finding was confirmed by the examination of cell proliferation and apoptosis, indicating the effectiveness of CSE and DMC in inhibiting the early stages of hepatocarcinogenesis by reducing cell proliferation and/or triggering apoptosis. Administration of CSE at a dose of 400 mg/kg induced cleaved caspase-3 expression in rats treated with DEN. However, administration of DMC at a dose of 10 mg/kg bw resulted in a significant reduction in the number of PCNA-positive cells and an increase in the expression of cleaved caspase-3 in rats treated with DEN. Several studies have shown that DMC can inhibit cell proliferation and cause apoptosis in different types of cells, including HeLa human cervical cancer cells [10], MCF-7 human breast cancer cells [27], A549 lung cancer cells [28], human pancreatic cancer cells [29], human colorectal carcinoma HCT116 and LOVO cells [30], as well as human hepatoma SMMC-7721 cells [31]. These findings suggested that DMC could inhibit the early stages of hepatocarcinogenesis by reducing cell proliferation and triggering apoptosis.

## 4. Materials and Methods

### 4.1. Chemicals

Diethylnitrosamine (DEN) and 3,3′-diaminobenzidine tetrahydrochloride hydrate (DAB) were purchased from Sigma-Aldrich (St. Louis, MO, USA). Rabbit polyclonal antibody to rat glutathione *S*-transferase placental form (GST-P) was purchased from MBL (Nagoya, Japan). Purified anti-human/mouse/rat PCNA antibody was obtained from Biolegend (San Diego, CA, USA). The Vectastain ABC kit was purchased from Vector Laboratories, Inc. (Burlingame, CA, USA).

### 4.2. Preparation of CSE and DMC

The fruit of *C. nervosum* was collected from the Lampang Agricultural Research and Development Center, Lampang, Thailand. This plant was identified by comparing voucher specimens of known identities (QGB7290, QGB17340, and QGB25139), which were deposited at the Queen Sirikit Botanic Garden, Chiang Mai, Thailand. The *C. nervosum* seed extract (CSE) was prepared as previously described [8]. Briefly, *C. nervosum* seeds were split up and dried at 40 °C in a hot air oven. The dried seed powder was soaked in dichloromethane, and CSE was obtained. The content of DMC in CSE was investigated using high-performance liquid chromatography (HPLC), according to Chariyakornkul et al. [8]. CSE was analyzed using reverse-phase HPLC with a Zorbax Eclipse Plus C18 column (4.6 × 250 mm, inner diameter 5 µm). The mobile phase consisted of mobile phase A (water) and mobile phase B (methanol), with a gradient elution and a flow rate of 1.0 mL/min. DMC was monitored at 340 nm.

CSE was applied to silica gel column chromatography and eluted with a gradient mixture of *n*-hexane and EtOAc (100:0 to 80:20). The DMC fractions were collected, and DMC was purified by crystallization with EtOAc-*n*-hexane. The isolated DMC, orange powder, was identified using ^1^H- and ^13^C-NMR. A gram of CSE contained 48.8 ± 18.0 mg of DMC, as measured by HPLC analysis. The ^1^H-NMR spectrum of DMC (500 MHz, δ, ppm, J/Hz) was as follows: 2.13 (3H, s, 5′CH_3_), 2.16 (3H, s, 3′-CH_3_), 3.66 (3H, s, 6′-CH_3_), 5.43 (1H, s, 4′-OH), 7.41 (3H, m, H-3, 4, 5), 7.65 (2H, m, H-2, 6), 7.84 (1H, d, *J* = 15.6 Hz, H_β_), 7.99 (1H, d, *J* = 15.6 Hz, H_α_), 13.60 (1H, s, 2′-OH) (Figure S1). The ^13^C NMR spectrum of DMC (125 MHz, δ, ppm) was as follows: 7.70 (5′-CH_3_), 8.39 (3′-CH_3_), 62.52 (6′-OCH_3_), 106.71 (C-1′), 109.03 (C-5′), 126.85 (C_α_), 128.57 (C-3, 5), 129.08 (C-2, 6), 130.36 (C-4), 135.50 (C-1), 143.05 (C_β_), 159.01 (C-6′), 159.37 (C-4′), 162.19 (C-2′), 193.53 (C=O) (Figure S2).

### 4.3. Animals and Experimental Protocol

Five-week-old male Wistar rats were purchased from the Nomura Siam International Co., Ltd. (Bangkok, Thailand). The animals were acclimatized to standard laboratory conditions, including a temperature of 21 ± 1 °C, a humidity of 50 ± 10%, 12 h of light and 12 h of dark, and were housed in a filter-top cage. Rats were allowed free excess to a pellet diet and drinking water throughout the experiment. The experimental protocol was approved by the Animal Care and Use Committee, Chiang Mai University (Protocol number: 2565/RT-0006).

**Figure 5:**
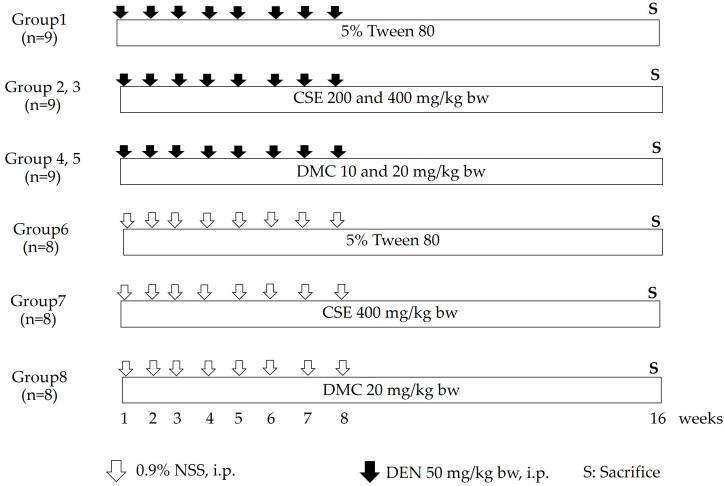
A schematic overview of the experimental design for studying the cancer chemopreventive effects of CSE and DMC on the DEN-induced early stages of hepatocarcinogenesis in rats. Diethylnitrosamine (DEN); normal saline solution (NSS); *C. nervosum* seed extract (CSE); 2′,4′-dihydroxy-6′-methoxy-3′,5′-dimethylchalcone (DMC).

The potential chemopreventive effects of CSE and DMC in rats were investigated using a DEN-induced early-stage hepatocarcinogenesis model. The experimental protocol is shown schematically in Figure 5. Male Wistar rats were divided into 8 groups. DEN 50 mg/kg bw was intraperitoneally injected into the rats in groups 1 to 5 once a week for 8 weeks, while NSS was intraperitoneally injected into the rats in groups 6 to 8. Throughout the experiment, rats in groups 1 and 6 were orally fed with non-toxic 5% Tween 80 as a vehicle control, whereas other groups were orally fed with CSE and DMC at low and high doses. At the end of the experiment, rats were sacrificed under anesthesia, and blood was collected for serum biochemical analysis. The liver, spleen, and kidney were excised and weighed. The liver was dissected into three pieces for immunohistochemical studies. One piece of liver tissue was kept at −80 °C until use for protein expression analysis. The remaining tissue was kept in 10% neutral buffered formalin for histopathological examination.

### 4.4. Biochemical Analysis

The biochemical activities of serum parameters such as ALT and AST were investigated, along with the levels of total protein, albumin, BUN, creatinine, total bilirubin, and direct bilirubin, which were measured with automated routine laboratory methods at Veterinary Diagnostic Center Company Limited, Chiang Mai, Thailand.

### 4.5. Histopathological Examination

Tissue sections of the liver and nodular lesions were fixed in 10% neutral buffered formalin and embedded in paraffin blocks. Paraffin sections with 4 µm thickness were stained with hematoxylin and eosin (H&E) for histopathological examination. A total of ten random low-power microscopic fields (40× magnification) were examined in each piece of liver. These fields were carefully evaluated by an impartial veterinarian who was unaware of the experimental groups in order to identify and confirm any histological abnormalities according to published criteria [32,33,34,35]. The numbers of focal hepatocellular hyperplasia, hepatocellular adenoma, and hepatocellular carcinoma per rat were reported.

### 4.6. Immunohistochemical Investigation

The immunohistochemical staining of glutathione *S*-transferase placental form (GST-P) and PCNA was determined by the method of Thumvijit et al. [36]. Briefly, the liver sections were deparaffinized and rehydrated with xylene and ethanol, respectively. To inhibit pseudo-peroxidase and inactivate non-specific protein binding, the slides were soaked in 3% H_2_O_2_ and 1% skimmed milk, respectively. The liver sections were incubated with rabbit polyclonal anti-rat GST-P antibody for GST-P staining and monoclonal mouse anti-rat PCNA antibody for PCNA staining, followed by biotin-conjugated secondary antibody. Subsequently, the sections were incubated in DAB solution and counterstained with hematoxylin. The number and area of GST-P-positive foci with a diameter greater than 0.2 mm were recorded using the LAS Interactive measurement program (Leica Microsystems, Wetzlar, Germany), whereas the number of PCNA-positive hepatocytes was counted in at least 30 fields per liver section under a light microscope.

### 4.7. Terminal Deoxynucleotidyl Transferase dUTP Nick-End Labeling (TUNEL) Assay

Apoptotic cells in liver sections were measured using the ApopTaq peroxidase in situ apoptosis detection kit according to the instruction manual. Briefly, the deparaffinized and dehydrated liver sections were treated with proteinase K and 3% H_2_O_2_, respectively. The sections were then incubated with equilibrium buffer and the working-strength terminal deoxynucleotidyl transferase (TdT) enzyme. After that, stop/wash buffer and an anti-digoxigenin antibody were added. The TUNEL-positive cells in liver sections were developed by soaking in DAB solution and counterstained with methyl green. The number of TUNEL-positive hepatocytes was counted in at least 30 fields per liver section under a light microscope.

### 4.8. Protein Expression Analysis by Western Immunoblotting

Frozen non-nodular liver tissues were homogenized with ice-cold radioimmunoprecipitation assay (RIPA) buffer containing protease inhibitors. The total protein was determined by Coomassie plus the better Bradford assay kit. An equal amount of pool sample was separated by electrophoresis in 10% SDS-PAGE and transferred to a nitrocellulose blotting membrane. After blocking with 5% skimmed milk in TPBS, the membranes were incubated with antibodies against GSTP (1:2000), cleaved caspase-3 (1:1000), or PCNA (1:2000) overnight at 4 °C. After that, the membranes were incubated with horseradish peroxidase-conjugated goat anti-rabbit IgG or rabbit anti-mouse IgG secondary antibodies for 1 h at room temperature. The protein visualization utilized chemiluminescence-based development approaches, with image acquisition performed utilizing the iBright™ CL-1500 imaging system (Thermo Fisher Scientific, Waltham, MA, USA). The protein loading was verified to be equal by stripping each membrane and reprobing it with an anti-β-actin antibody (1:50,000). Band density levels were analyzed using the “Measure” function in ImageJ 1.410 software.

### 4.9. Statistical Analysis

Results were expressed as mean ± SD. Statistical analysis comparing groups was conducted using a one-way analysis of variance (ANOVA) with a Bonferroni post hoc test, except for the results of cell proliferation and apoptosis, which were analyzed using a post hoc least-significant difference test. Statistical significance was defined as a value of *p* < 0.05.

## 5. Conclusions

In conclusion, 2′,4′-Dihydroxy-6′-methoxy-3′,5′-dimethylchalcone (DMC), which is one of the major compounds derived from CSE, may be a potential chemopreventive agent in *C. nervosum* seeds. The activity of DMC targeted the early stages of hepatocarcinogenesis by promoting cell apoptosis and inhibiting cell proliferation. Furthermore, DMC is not carcinogenic to rats. Thus, this compound might have the potential to enhance the effectiveness of cancer prevention strategies.

## Figures and Tables

**Table 1:** Effects of CSE and DMC on body weight and consumption of diet and water in rats.

Group	Treatments	Body Weight (g)		Consumption (per Rat per Day)	
		Initial	Final	Diet (g)	Water (mL)
1	DEN	211 ± 9	468 ± 27 *	19.5 ± 1.1 *	28.8 ± 4.8
2	DEN + CSE 200 mg/kg bw	212 ± 20	490 ± 60	20.8 ± 1.4	36.9 ± 7.4 **
3	DEN + CSE 400 mg/kg bw	212 ± 17	451 ± 49	19.6 ± 1.2	31.8 ± 5.9
4	DEN + DMC 10 mg/kg bw	211 ± 7	465 ± 15	19.2 ± 1.3	31.1 ± 6.3
5	DEN + DMC 20 mg/kg bw	211 ± 5	459 ± 31	19.1 ± 1.5	37.0 ± 9.5 **
6	NSS	212 ± 8	561 ± 26	22.3 ± 1.2	35.4 ± 4.4
7	NSS + CSE 400 mg/kg bw	211 ± 15	494 ± 49 *	20.3 ± 1.3 *	38.2 ± 5.9
8	NSS + DMC 20 mg/kg bw	212 ± 13	533 ± 35	21.5 ± 1.1	34.3 ± 4.9

The results are presented as mean ± SD. Diethylnitrosamine (DEN); normal saline solution (NSS); *C. nervosum* seed extract (CSE); 2′,4′-dihydroxy-6′-methoxy-3′,5′-dimethylchalcone (DMC). * Significantly different compared to the NSS-treated group (*p* < 0.05). ** Significantly different compared to the DEN-treated group (*p* < 0.05).

**Table 2:** Effects of CSE and DMC on relative internal organ weights.

Group	Treatments	Relative Organ Weight (%)		
		Liver	Spleen	Kidney
1	DEN	2.64 ± 0.16	0.17 ± 0.02	0.56 ± 0.06
2	DEN + CSE 200 mg/kg bw	2.74 ± 0.17	0.19 ± 0.08	0.58 ± 0.07
3	DEN + CSE 400 mg/kg bw	2.88 ± 0.18	0.16 ± 0.01	0.63 ± 0.03 **
4	DEN + DMC 10 mg/kg bw	2.63 ± 0.15	0.16 ± 0.01	0.58 ± 0.03
5	DEN + DMC 20 mg/kg bw	2.67 ± 0.35	0.17 ± 0.01	0.58 ± 0.03
6	NSS	2.60 ± 0.13	0.14 ± 0.02	0.53 ± 0.05
7	NSS + CSE 400 mg/kg bw	2.90 ± 0.16	0.14 ± 0.01	0.64 ± 0.01 *
8	NSS + DMC 20 mg/kg bw	2.53 ± 0.20	0.13 ± 0.01	0.54 ± 0.03

The results are presented as mean ± SD. Diethylnitrosamine (DEN); normal saline solution (NSS); *C. nervosum* seed extract (CSE); 2′,4′-dihydroxy-6′-methoxy-3′,5′-dimethylchalcone (DMC). * Significantly different compared to the NSS-treated group (*p* < 0.05). ** Significantly different compared to the DEN-treated group (*p* < 0.05).

**Table 3:** Effects of CSE and DMC on serum biochemical parameters in rats.

Group	Treatments	Biochemical Parameters					
		Total Protein (g/dL)	Albumin (g/dL)	Total Bilirubin (mg/dL)	Direct Bilirubin (mg/dL)	BUN (mg/dL)	Creatinine (mg/dL)
1	DEN	7.27 ± 0.22	3.94 ± 0.10	0.11 ± 0.01	0.07 ± 0.01 *	20.0 ± 1.0 *	0.73 ± 0.03
2	DEN + CSE 200 mg/kg bw	7.30 ± 0.18	3.96 ± 0.05	0.10 ± 0.02	0.07 ± 0.02	20.1 ± 0.5	0.72 ± 0.02
3	DEN + CSE 400 mg/kg bw	7.61 ± 0.21	4.14 ± 0.05 **	0.11 ± 0.01	0.07 ± 0.01	18.4 ± 0.9	0.71 ± 0.04
4	DEN + DMC 10 mg/kg bw	7.16 ± 0.05	3.94 ± 0.14	0.08 ± 0.01 **	0.05 ± 0.01 **	18.1 ± 1.0 **	0.67 ± 0.02 **
5	DEN + DMC 20 mg/kg bw	7.26 ± 0.15	3.94 ± 0.13	0.09 ± 0.02	0.05 ± 0.01 **	20.2 ± 0.6	0.71 ± 0.02
6	NSS	7.23 ± 0.23	3.87 ± 0.10	0.09 ± 0.01	0.05 ± 0.01	18.0 ± 0.6	0.68 ± 0.03
7	NSS + CSE 400 mg/kg bw	7.10 ± 0.13	3.77 ± 0.05	0.08 ± 0.01	0.04 ± 0.01	19.0 ± 0.4	0.63 ± 0.02
8	NSS + DMC 20 mg/kg bw	6.92 ± 0.23	3.65 ± 0.14 *	0.09 ± 0.01	0.05 ± 0.01	18.9 ± 1.2	0.64 ± 0.03

The results are presented as mean ± SD. Diethylnitrosamine (DEN); normal saline solution (NSS); *C. nervosum* seed extract (CSE); 2′,4′-dihydroxy-6′-methoxy-3′,5′-dimethylchalcone (DMC). * Significantly different compared to the NSS-treated group (*p* < 0.05). ** Significantly different compared to the DEN-treated group (*p* < 0.05).

**Table 4:** Effects of CSE and DMC on histopathological lesions in rat livers.

Group	Treatments	The Number of Histopathological Lesions per Rat		
		Focal Hepatocellular Hyperplasia	Hepatocellular Adenoma	Hepatocellular Carcinoma
1	DEN	4.33 ± 2.66 *	3.13 ± 2.85	1.50 ± 1.20
2	DEN + CSE 200 mg/kg bw	2.14 ± 3.67	1.86 ± 2.85	0.57 ± 0.98
3	DEN + CSE 400 mg/kg bw	0.25 ± 0.46 **	0.75 ± 1.16	0.56 ± 0.53
4	DEN + DMC 10 mg/kg bw	1.25 ± 1.58	3.00 ± 3.12	0.75 ± 1.39
5	DEN + DMC 20 mg/kg bw	2.86 ± 4.63	4.88 ± 4.91	3.00 ± 5.21
6	NSS	0.00 ± 0.00	0.00 ± 0.00	0.00 ± 0.00
7	NSS + CSE 400 mg/kg bw	0.00 ± 0.00	0.00 ± 0.00	0.00 ± 0.00
8	NSS + DMC 20 mg/kg bw	0.00 ± 0.00	0.00 ± 0.00	0.00 ± 0.00

The results are presented as mean ± SD. Diethylnitrosamine (DEN); normal saline solution (NSS); *C. nervosum* seed extract (CSE); 2′,4′-dihydroxy-6′-methoxy-3′,5′-dimethylchalcone (DMC). * Significantly different compared to the NSS-treated group (*p* < 0.05). ** Significantly different compared to the DEN-treated group (*p* < 0.05).

## Data Availability

The original contributions presented in the study are included in the article/Supplementary Material; further inquiries can be directed to the corresponding author.

## Supplementary Materials

The following supporting information can be downloaded at: https://www.mdpi.com/article/10.3390/plants13141975/s1, Figure S1: ^1^H-NMR (500 MHz, CDCl_3_) spectrum of 2′,4′-dihydroxy-6′-methoxy-3′,5′-dimethylchalcone; Figure S2: ^13^C-NMR (125 MHz, CDCl_3_) spectrum of 2′,4′-dihydroxy-6′-methoxy-3′,5′-dimethylchalcone.
