# Supplementary material for: Cancer Chemopreventive Effect of 2′,4′-Dihydroxy-6′-methoxy-3′,5′-dimethylchalcone on Diethylnitrosamine-Induced Early Stages of Hepatocarcinogenesis in Rats
Source: Plants (Basel). 2024 Jul 19;13(14):1975. doi: 10.3390/plants13141975 (PMC11280862; doi:10.3390/plants13141975)
Supplement: Supplementary file 1 [file plants-13-01975-s001.zip › plants-3079761-supplementary.pdf]

# Supplemental Material

## Outline

*Figure S1*..........................................................................................................................3

*Figure S2*..........................................................................................................................4

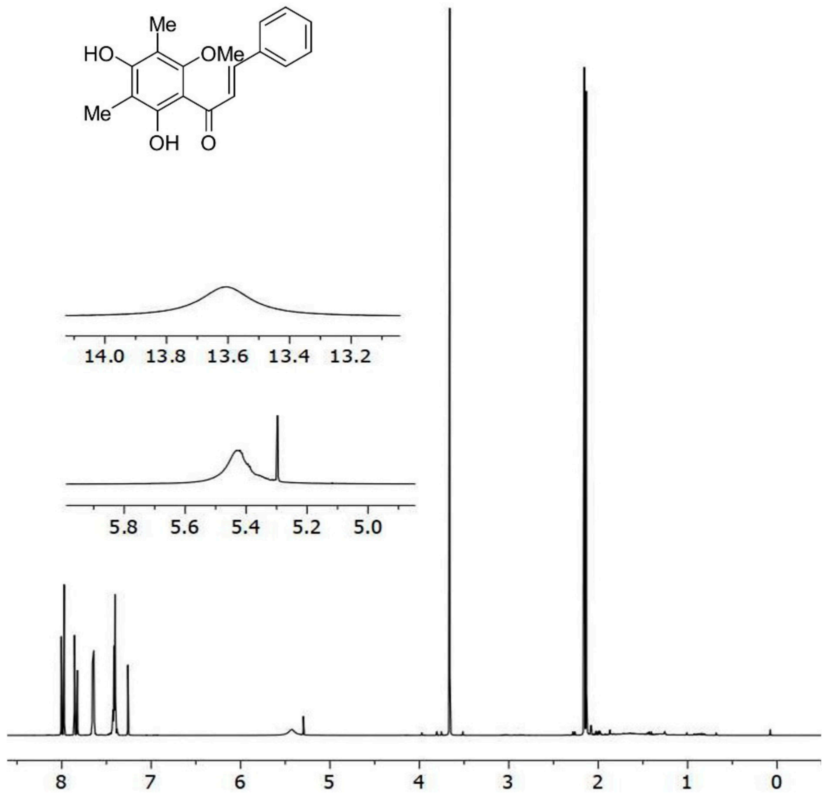


Figure S1: $^{1}$H-NMR (500 MHz, $CDCl_3$) spectrum of 2′,4′-dihydroxy-6′-methoxy-3′,5′-dimethylchalcone

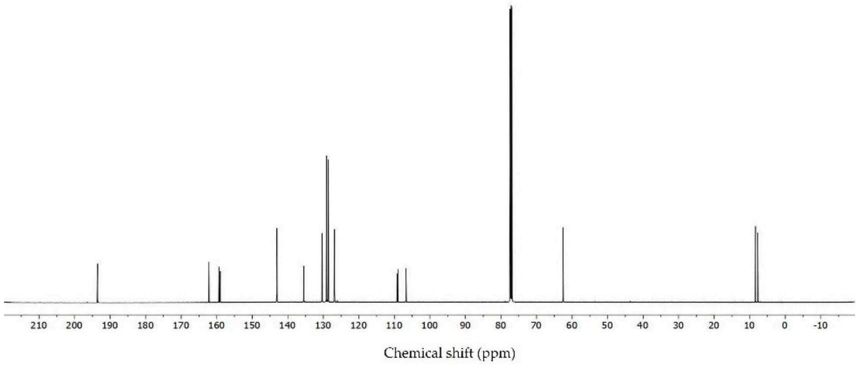


Figure S2: $^{13}C$-NMR (125 MHz, $CDCl_3$) spectrum of 2´,4´-dihydroxy-6´-methoxy-3´,5´-dimethylchalcone.
